# ESPEE: Event-Based Sensor Pose Estimation Using an Extended Kalman Filter

**DOI:** 10.3390/s21237840

**Published:** 2021-11-25

**Authors:** Fabien Colonnier, Luca Della Vedova, Garrick Orchard

**Affiliations:** 1 Temasek Laboratories, National University of Singapore, Singapore 117411, Singapore; tslgmo@nus.edu.sg; 2 Institute for Infocomm Research, A*STAR, Singapore 138632, Singapore; 3 Open Source Robotics Corporation, Singapore 138633, Singapore; luca@openrobotics.org

**Keywords:** event-based sensor, visual odometry, extended Kalman filter, computer vision, structureless measurement model

## Abstract

Event-based vision sensors show great promise for use in embedded applications requiring low-latency passive sensing at a low computational cost. In this paper, we present an event-based algorithm that relies on an Extended Kalman Filter for 6-Degree of Freedom sensor pose estimation. The algorithm updates the sensor pose event-by-event with low latency (worst case of less than 2 μs on an FPGA). Using a single handheld sensor, we test the algorithm on multiple recordings, ranging from a high contrast printed planar scene to a more natural scene consisting of objects viewed from above. The pose is accurately estimated under rapid motions, up to 2.7 m/s. Thereafter, an extension to multiple sensors is described and tested, highlighting the improved performance of such a setup, as well as the integration with an off-the-shelf mapping algorithm to allow point cloud updates with a 3D scene and enhance the potential applications of this visual odometry solution.

## 1. Introduction

Six Degree of Freedom (6-DoF) poses information that is useful in many applications, most notably in the emerging fields of Augmented Reality and Virtual Reality (AR/VR), and also in autonomous mobile vehicles. In AR/VR, 6-DoF viewpoint tracking is required for accurate rendering on a head-mounted display [1]. In the realm of mobile vehicles, knowledge of a vehicle’s 6-DoF pose is required in motion control [2], as well as in Simultaneous Localization and Mapping (SLAM) algorithms [3,4]. Furthermore, full 6-DoF pose information is especially useful for aerial vehicles whose motion is far less constrained than ground vehicles.

An ideal 6-DoF pose estimation sensor would not rely on any pre-installed infrastructure in the environment. The sensor would be a self-contained, embedded system suitable for deployment in unknown environments. However, pose estimation typically requires the sensor to have either a prior or acquired knowledge of its environment. Active depth sensors greatly simplify the problem of estimating the structure of the environment, but also come with the disadvantage of requiring additional power for emission, high cost (in the case of LiDAR), and risking cross-talk between sensors which co-exist in the same environment. Visual odometry is a possible passive sensing approach for pose estimation [5].

However, for applications such as AR/VR and aerial vehicles, latency is also a concern. Sensing latencies complicate closed-loop motion control, and AR/VR applications target motion-to-photon latencies [6] in the order of 10–20 ms [7], which is similar to the latency required to obtain data from a frame-based sensor. Thus, relying on a frame-based sensor leaves little to no time for processing, rendering, and updating the display.

Event-based vision sensors [8] offer a potential solution to overcome the latency problem. They are compact passive low-power (10 mW) vision sensors that relate information about the environment with low latency (typically ranging from 10 μs to 1 ms [9]). Event-based vision sensors also provide very sparse data, allowing for fast and efficient computation, even in embedded systems [6,10,11,12,13,14]. These sensors provide data as a stream of “events”, which is very different from the standard computer vision “frame” data format, and therefore, processing event data requires the design of new algorithms. Encouraged by the desirable properties of event-based vision sensors, many researchers have taken up the task, and new solutions have recently been proposed [15].

Several recent works show that frames can be recreated from events in pose estimation applications [16,17,18] or for this sole purpose [19,20,21], thereby opening the door for existing computer vision algorithms to be used for processing. However, the additional latency and processing costs associated with recreating frames negates the low-latency, low-power benefits of the event-based sensor. Nevertheless, these works indicate that the information present in frame-based videos is also present in a stream of events and can be extracted by designing the right algorithm.

In this paper, we present a real-time approach for Event-based Sensor Pose Estimation using an Extended Kalman Filter (ESPEE). As the name suggests, ESPEE estimates the 6-DoF pose of one or an array of sensors based solely on visual information. The novelty lies in the event-by-event pose estimation processing, which requires neither intensity gradient information nor direct depth measurements. The algorithm uses an original event-to-map association to compute the pose estimation. The map of the environment is expressed as a list of 3D points.

Although ESPEE can handle an arbitrary 3D map, we initially use a planar (or near planar) scene assumption in the monocular experiments to simplify the map generation process. This assumption is reasonable for a downward-facing sensor on an aerial vehicle. Finally, an implementation of the EMVS mapping algorithm [22] is combined with the localization algorithm to demonstrate a complete visual odometry application with two sensors.

Experiments with a single sensor and two sensors placed orthogonally are performed to display the accuracy of ESPEE for the different configurations. The recordings show the ability of the algorithm to estimate pose with low latency. Finally, a comparison is made between one sensor, two sensors, and two sensors with mapping on the same recording.

### 1.1. Event-Based Vision Sensors

Event-based vision sensors [8], also referred to as silicon retinas and Dynamic Vision Sensors (DVSs), consist of an array of independently operating pixels, which detect and report when their illumination has changed by more than a threshold percentage (or equivalently, when their log-illumination has changed by more than a fixed threshold), over a wide intra-scene dynamic range (140 dB [23]). The thresholds for increases and decreases in intensity can be set individually by the user.

The event-based sensor provides a sparse output, consisting of a stream of events, where each event consists of an integer *x–y* pixel address and 1 bit indicating the direction of the change. The low latency of the sensor readout allows approximating the time of the change detection as the time at which the event was received from the sensor. The device receiving the events from the sensor typically has an onboard clock that is used to attach a timestamp to each received event, thus allowing the recorded event stream to be replayed later with accurate timing. In this work, we do not use the timestamps or polarities, only the *x* and *y* addresses.

**Figure 1:**
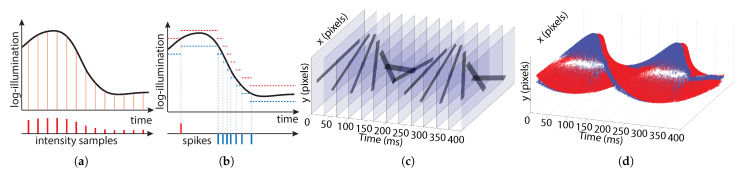
Comparison between frame-based and event-based vision sensor principles. (**a**) Sampling of a frame-pixel occurs at constant time intervals. (**b**) Sampling of an event pixel occurs whenever the log-intensity change exceeds a threshold. The ON and OFF thresholds are represented with red and blue dotted lines, respectively, and updated at each event. (**c**) Example of 24 fps frame-data (simulated) that would be generated by a rotating bar. (**d**) Example of event data gathered when viewing the same rotating bar (actual recording). Red and blue dots show the ON and OFF events capturing the contrast of the bar’s edges relative to the background according to time.

Figure 1 shows the difference in data acquisition between frame-based and event-based sensors. Frame-based pixels sample intensity at constant time intervals (Figure 1a). The frame-timing is predetermined. Information about the scene is encoded in the intensity values contained in each pixel sample. Event-based pixels sample time at constant log-intensity intervals (Figure 1b). Each sample consists of a single bit (polarity) indicating whether an increase (red) or decrease (blue) in intensity occurred. Information is encoded in the timing of the samples.

Figure 1c shows simulated data for a 24 frames-per-second (fps) frame-based sensor viewing a rotating black bar. The majority of the data in each frame is redundant (indicated by translucent regions). Figure 1d shows actual data from a rotating bar recording for an event-based sensor. Red and blue events indicate increases and decreases in intensity, respectively. Redundant background information is not acquired, but the location of the bar can still be detected.

### 1.2. Related Event-Based Algorithms for Visual Odometry

One of the earliest works on event-based pose estimation [24] used an event-based particle filter to track the 2-DoF location of an indoor robot by using an upward-facing sensor viewing a precomputed map of the ceiling. The approach was later extended to handle 3-DoF motion (allowing the robot to rotate) and to compute the ceiling map on the fly [25]. The approach was further modified to handle 6-DoF motion and mapping but relied on augmenting the event-stream with depth information obtained from an RGB-D sensor [26].

In the same year, an event-based particle filtering approach was proposed for estimating the 3-DoF rotation of a sensor and creating a mosaic of the scene [16]. Restricting the sensor to pure rotational motion removes the need to calculate or rely on any depth information. The same authors later proposed an Extended Kalman Filter (EKF) approach to 3D mapping and 6-DoF pose tracking [27]. The approach achieved impressive results but relied on a powerful GPU to accelerate the algorithm to real-time performance.

The authors of [28] proposed an event-based method for tracking the relative 6-DoF pose between an event-based sensor and an object in the world. The approach required accurate initialization and a precomputed model of the object. Each time an event is generated by the sensor, it is backprojected to generate a corresponding 3D ray in the world, which is matched to the closest edge on the object based on the algorithm’s current estimate of the object’s pose. The object’s 3D orientation and 3D translation are then each updated to reduce the distance between the ray and the matched point. The amount by which the orientation and translation are updated is controlled by separate hand-tuned gain parameters, which must be set before running the algorithm.

A visual odometry approach was proposed for estimating 6-DoF sensor location and building a 3D map [18]. The approach accumulates events to generate binary images at high rates (order of 1000 fps). For each new binary image, a reference image is generated based on the latest sensor pose and a keyframe for which depth has already been computed. The algorithm then computes the incremental pose update, which minimizes the error between the new binary image and the reference image. Although the approach uses frames created from events, it is capable of calculating over 500 pose updates per second while simultaneously generating a 3D map of the environment.

Later, the same group proposed a 6-DoF pose tracking approach based on Bayesian Filtering [29]. The algorithm computes pose updates event by event and explicitly accounts for outliers and noise, but it relies on a precomputed photometric depth map of the scene and can only run real-time at very low event rates.

Approaches such as [28] keep track of only one possible pose but require the tuning of gain parameters to match the expected range of motion in the scene. The need to tune parameters is overcome by particle filtering approaches, which keep track of many possible gain parameter values in parallel and select the best result. However, to track a wide range of possible values for the gain parameters, many particles must be used, thereby driving up the computational requirements. The EKF and Bayesian filtering approaches instead keep track of the uncertainty in the pose and explicitly compute the gain that should be used. In the case of our EKF, explicitly computing the gain reduces both the memory and computational requirements compared to a particle filter.

## 2. Espee Algorithm

The ESPEE algorithm computes pose estimation based on a single or several event-based sensors. To do so, the first step consists of data processing to reduce noise. This part is optional as it depends on the data quality, which can be improved by tuning the sensor parameters before the recording. As ESPEE relies on a point cloud as a map, Section 2.2 explains how it is initialized. The two main parts of the algorithm are the association of each incoming event with a point of the map reprojected on the sensor frame at the estimated pose (Section 2.3) and the EKF computation, which minimizes the difference between the event pixel and the 3D point previously associated with estimating the pose (Section 2.4). All the EKF parameters used for each test are summed up in Table A1 in Appendix A. Finally, Section 2.6 explains how the point cloud is updated in the different experiments.

### 2.1. Preprocessing

The event data is preprocessed to remove noise, limit the firing rate of “hot" pixels, and remove lens distortion.

A background activity filter [14,30] removes isolated noise events by comparing each event’s timestamp to the ones of the most recent events captured by the neighboring pixels. If none of the neighboring pixels have generated events recently (within a threshold time period), the event is considered to be an isolated noise event and is discarded.

For event-based sensors, “hot” pixels are pixels generating events at a fast rate regardless of the visual stimulus. These “hot” pixels are suppressed by implementing a refractory period, which limits the maximum event rate per pixel (and therefore the maximum event rate for the whole sensor).

Lens distortion is also compensated for before running the other steps of the algorithm. The lens distortion parameters are obtained thanks to the open-source ROS camera calibration package [31], relying partly on [32], by capturing grayscale images.

### 2.2. 3D Point Cloud Initialization

To initialize the point cloud that will be used for the pose estimation, a given number of events, typically $n_{i}=2000$ for the DAVIS240C, is accumulated. Similar to [18,28], any point in the world that generates events is used by the algorithm. Each event is then backprojected to the parallel plane to the focal one at distance *d* to generate the initial list of 3D points, as the sensor is assumed to be static during the initialization. Theoretically, the depth *d* is unitless because the world is only defined up to scale. In our implementation, this depth *d* is an initial parameter to scale the displacement of the sensor, and therefore, allows translation comparison with the ground truth, which would only be proportional otherwise.

**Figure 2:**
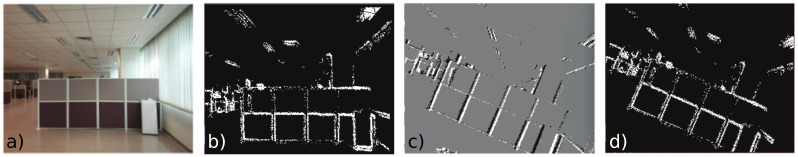
Example of point cloud initialization and LUT computation. (**a**) A photo of the scene captured by a cell phone camera (here only for illustration and not used in the processing). (**b**) Frame showing the accumulated events used to generate the point cloud at initialization. The 3D points are the projection of the events onto a plane at a distance provided by the user. (**c**) A frame of events captured during a sensor rotation. (**d**) The same 3D points initialized in (**b**), projected into the LUT for a later sensor pose corresponding to (**c**).

Figure 2 shows an example scene (Figure 2a is given as a reference for the reader and is not used in the algorithm) with the corresponding initialization map (Figure 2b). After rotation of the sensor, the scene is viewed from a different angle (Figure 2c). If the pose is accurately tracked, the change in the appearance of the scene should be accurately reflected when the 3D point cloud is reprojected on the frame (Figure 2d). This reprojection is later referred ti as a Look-Up Table (LUT).

### 2.3. Pixel to 3D Point Association

Similarly to [28], matching an event to a 3D point is performed based on the distance to its reprojected counterpart in the image plane.

By projecting the 3D point cloud onto the image plane using the current estimate of sensor pose, a Look-Up Table (LUT) the size of the image is generated containing the inverse depth:

(1) $$\mathrm{LUT}\left(x,y\right)=\frac{1}{d}\;\mathrm{where}\;x<n_{x},\;y<n_{y}\;\mathrm{and}\;\left[x,y\right]\in N^{2},$$

where LUT(x,y) is the lookup table value for pixel ${\left[x,y\right]}^{T}$, *d* is the depth of the 3D point in camera coordinates, and $n_{x},n_{y}$ is the frame dimension of the sensor. When no point is reprojected to a given pixel location, no information should be registered. In the code, a value of zero indicates this state.

**Figure 3:**
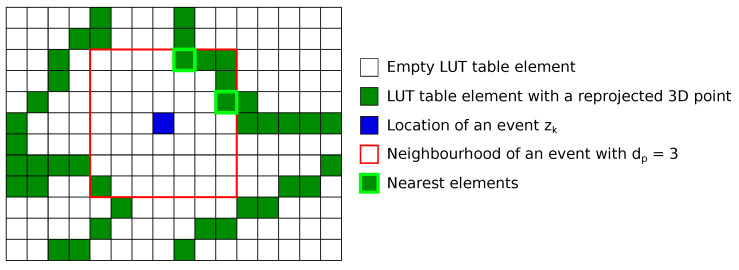
Description of the pixel association process once the LUT is computed. For each event, a search of the closest map element is performed in a given neighborhood. In this example, the LUT-pixel $h_{k}$ to be used for localization will be chosen at random between the two nearest elements.

For each incoming event $z_{k}^{\left(p\right)}={\left[x_{k},y_{k}\right]}^{T}$, where $z_{k}^{\left(p\right)}$ is the *k*-th event in pixel coordinates ${}^{\left(p\right)}$ and occurs at pixel ${\left[x_{k},y_{k}\right]}^{T}$, we search the LUT in a neighborhood around $z_{k}^{\left(p\right)}$ for possible LUT-pixel matches. Figure 3 illustrates an example of a pixel association. If no match is assigned, we move on to processing the next event. If at least one LUT pixel is found in the event’s neighborhood, the match with the nearest pixel location to $z_{k}^{\left(p\right)}$ is used. If there is a tie for the nearest LUT-pixel, one of the candidates is selected at random to become the matching LUT-pixel $h_{k}^{\left(p\right)}$. We typically restrict the neighborhood to a region lying within $d_{p}=3$ pixels of the event.

The ESPEE algorithm assumes that sensor motion will not cause the projection of a 3D point to move by more than $d_{p}$ pixels before the LUT is refreshed. As a 1 kHz LUT refresh rate is easily achieved on a CPU, this assumption holds theoretically for translation and rotation above 10 m·s^−1^ (with 0.5 m scene depth) and 1000 deg·s^−1^, with the DAVIS240C sensor and 4 mm focal length lens for $d_{p}=5$.

### 2.4. Pose Estimation with the Ekf

#### 2.4.1. Definition

The state vector used describes only the pose of the sensor or the rig in the case of multiple sensors. The state is thus $X={\left[\begin{array}{cc} p & q \end{array}\right]}^{T}$, where *p* and *q* are the position vector and the quaternion describing the transformation from local to world coordinate, respectively. In this work, an Extended Kalman Filter represents the uncertainties of the estimated state vector via the covariance matrix P of the corresponding state error vector δX:

(2) $$\begin{array}{ccc} P & = & cov(\delta X)\\ \delta X & = & {\left[\begin{array}{cc} \delta p & \delta \phi \end{array}\right]}^{T} \end{array}$$

where P is initialized as a diagonal matrix. δp and δϕ are vectors representing the position and rotation errors, respectively. δϕ defines a rotation of angle ||δϕ|| about the unit vector defined as δϕ/||δϕ|| [33]. As *q* and δϕ are four and three-dimension vectors, respectively, X is a seven-dimension vector, whereas δX and later its discretized version ΔX are six-dimension vectors.

The error quaternion δq is obtained from the rotation vector δϕ through the map ζ defined as:

(3) $$\begin{array}{ccc} \delta q & = & \zeta (\delta \phi )\\ \zeta :vs.\mapsto \zeta (v) & = & \left[\begin{array}{c} \cos(\frac{1}{2}||v||)\\ \sin(\frac{1}{2}\left||v||\right)\frac{v}{\left||v|\right|} \end{array}\right] \end{array}$$

and the state quaternion is obtained thanks to the relation $q_{k+1}=\delta q_{k}⊗q_{k}$, where ⊗ is the quaternion multiplication operator.

#### 2.4.2. Prediction Step

As the EKF is processed event-by-event, the model assumes a constant pose between two events. This assumption holds for a given scene, and the event stream is directly proportional to the motion, i.e., the more the sensor moves, the more events are generated. Thus, this model assumes that uncertainty in position grows proportional to the number of events received instead of proportional to time passed. Moreover, we only used the events that are associated with a 3D point to filter noisy events. The prediction is then:

(4) $$\begin{array}{c} P_{k}^{-}=P_{k-1}^{+}+Q\\ X_{k}^{-}=X_{k-1}^{+} \end{array}$$

where the superscripts ${}^{+}$ and ${}^{-}$ indicate the a posteriori and a priori estimate, respectively. Q is the covariance matrix of the state error increment between two events. It is a constant diagonal matrix that can be tuned depending on the experimental setup (visual scene, illuminance, lens, sensor resolution, etc.). The bigger the value $Q_{ii}$ is compared to the other degrees of freedom, the more likely the EKF algorithm will minimize the error between the event pixel $z_{k}^{\left(p\right)}$ and the matching LUT-pixel $h_{k}^{\left(p\right)}$ by modifying this given degree of freedom. Prior knowledge about the motion of the sensor can thus be built into Q (for example, $Q_{22}$, which corresponds to the *y*-axis sensor translation, can be kept very small for ground vehicles operating on flat ground with a forward-facing sensor).

#### 2.4.3. Update Step

The EKF module receives the most recent event, $z_{k}^{\left(p\right)}$, the closest matching LUT-pixel $h_{k}^{\left(p\right)}$ in pixel units, and the inverse depth 1/d from the pixel to 3D point association module. First, $z_{k}^{\left(p\right)}$ and $h_{k}^{\left(p\right)}$ are converted into focal plane units ${}^{\left(f\right)}$, denoted as $z_{k}^{\left(f\right)}={\left[u_{z},v_{z}\right]}^{T}$ and $h_{k}^{\left(f\right)}={\left[u_{h},v_{h}\right]}^{T}$. Then, the innovation $y_{k}$ is computed as follows when normalized by the focal length:

(5) $$y_{k}=z_{k}^{\left(f\right)}-h_{k}^{\left(f\right)}.$$

The measurement jacobian is defined as $H_{k}=\frac{\partial y_{k}}{\partial {\hat{x}}_{k}}$. As $y_{k}$ and ΔX are a pixel difference and a pose difference, respectively, it can be easily deduced that $H_{k}$ is equal to the image Jacobian (i.e., the matrix defined as the relation between camera motion and pixel motion) [34]. Thus, $H_{k}$ is expressed as follows, when normalized by the focal length:

(6) $$H_{k}=\left[\begin{array}{cccccc} -\frac{1}{d} & 0 & \frac{u_{h}}{d} & u_{h}v_{h} & -(1+u_{h}^{2}) & v_{h}\\ 0 & -\frac{1}{d} & \frac{v_{h}}{d} & 1+v_{h}^{2} & -u_{h}v_{h} & -u_{h} \end{array}\right].$$

The standard EKF equations can therefore be applied:

(7) $$\begin{array}{ccc} S_{k} & = & H_{k}P_{k}^{-}H_{k}^{T}+R\\ K_{k} & = & P_{k}^{-}H_{k}^{T}S_{k}^{-1}\\ \Delta X_{k} & = & K_{k}y_{k}\\ P_{k}^{+} & = & \left(I_{6}-K_{k}H_{k}\right)P_{k}^{-} \end{array}$$

where $S_{k}$ is the residual covariance, R is the predefined measurement noise covariance in focal plane units, $K_{k}$ is the Kalman Filter gain, $\Delta X_{k}$ is the resulting correction vector, and $I_{6}$ is the 6×6 identity matrix.

To get the state estimate $X_{k}^{+}$ from $\Delta X_{k}$, the following operations should be done:

(8) $$\begin{array}{c} p_{k}^{+}=p_{k}^{-}+\Delta p_{k}\\ q_{k}^{+}=\zeta \left(\Delta \phi_{k}\right)⊗q_{k}^{-} \end{array}$$

In the case of multiple sensors, only the Jacobian $H_{k}$ has to be modified to take into account the frame transformation. Indeed, the relative motion implied by the pixel position difference observed is relative to each sensor frame; the Jacobian has to be transposed in the rig frame, which has been chosen to be the first sensor frame. Thus, the new Jacobian $H_{n,k}$ for a given sensor *n* is expressed as follows:

(9) $$H_{n,k}^{T}=\left[\begin{array}{cc} R_{n,1} & t_{n,1}^{\times }R_{n,1}\\ 0 & R_{n,1} \end{array}\right]\cdot H_{k}^{T},$$

where $R_{n,1}$ and $t_{n,1}^{\times }$ are the rotation matrix and the skew-symmetric matrix of the translation vector $t_{n,1}$, respectively. $R_{n,1}$ and $t_{n,1}$ represent the transformation from the *n*-th sensor frame to the first one. Both are fixed and known initially as the rig is a rigid body.

### 2.5. Implementation

A floating-point precision implementation of ESPEE was written in C++. The LUT creation, the pixel to 3D point association, and EKF updates all occur on a single thread, but ESPEE still achieves real-time performance on several computational devices when one input sensor is used. The maximum sustained event processing rate for different devices was tested using a 1 kHz LUT update rate and a feature list of length 3500 (see Table 1).

A fixed precision version of the algorithm was also implemented on FPGA, processing 1.5 million events per second (Mevts/s). An FPGA makes it easy to parallelize the EKF algorithm and the LUT update, resulting in a LUT refresh rate over 2 kHz. The worst-case latency, when two events happen at the same time, between receiving the second event and finishing its associated pose update is under 2 μs.

### 2.6. Point Cloud Update

Two methods were used to update the 3D point cloud. The first one still assumes a planar scene and is used with a monocular sensor, whereas the second one uses the EMVS algorithm [22] and is used with the dual-sensor setup. During the monocular experiment, the point cloud is updated whenever the estimated pose is farther away from the previous keyframes. The first keyframe is established at the initialization of the algorithm. A new keyframe is triggered when the sensor position lies further than a threshold distance from all previous keyframe positions. The threshold is defined as a percentage of the mean scene depth (following the same strategy shown in [18] to trigger new keyframes). When a new keyframe is reached, we use the next $n_{i}$ events to add 3D points to the map. Any of the $n_{i}$ events that are successfully matched to existing 3D points are used to update the EKF pose as usual but do not get added to the point cloud (since corresponding 3D points for these events already exist). Events for which no matching 3D point is found are added to the map by backprojecting the event out into the world until it crosses the plane z=d in world coordinates.

Another method was used with the dual-sensor setup to avoid the planar assumption. The EMVS algorithm was used for both sensors individually, and each update replaces the previous data, as done in [18]. The output was fused into one point cloud. It should also be noticed that no 3D point refinement is made between keyframes.

The use of the EMVS algorithm was attempted with the monocular setup but did not yield a very stable solution, as large drifts were observed after 3–4 keyframes leading to inaccurate pose and thus inaccurate point cloud extraction.

## 3. Results

**Figure 4:**
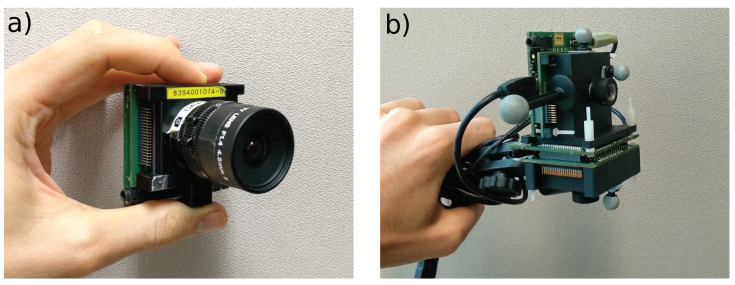
The two different sensor setups. (**a**) DAVIS240C sensor with a 4.5 mm lens used for the monocular experiments. (**b**) The dual sensor rig mounted on a tripod. Both sensors are equipped with low distortion 3.6 mm lenses, thanks to 3D printed mounts.

To quantitatively test ESPEE, two setups were used, one with a single sensor and another with two sensors placed orthogonally (see Figure 4). The monocular sensor setup demonstrates the ability of the algorithm to keep track of its pose. The two-sensor setup improves the localization and proves to be robust enough to be coupled with the EMVS mapping algorithm [22]. We compared ESPEE pose estimates to pose measurements made by a Vicon motion capture system (http://www.vicon.com/, accessed on 25 November 2021) composed of 14 cameras running at 200 Hz. All the recordings made, with their results, are presented in the videos available as Supplementary Materials.

### 3.1. Monocular Experiments

The experiments were performed with a handheld sensor moving over three different visual scenes. The first scene used strong black stimuli printed on white paper lying on the floor. We call this the Black and White Planar test, “BW Planar”. The second scene consisted of objects lying on the floor, viewed from approximately 1.2 m high. This scene has fewer contrasts than the BW Planar scene and has some variations in-depth. We call this second test “Objects”. A third test, named “Lines”, is performed over a uniform background with non-crossing black lines. This test is designed to demonstrate the robustness of the localization when only edges and no corners can be found in the map.

#### 3.1.1. Handheld Experiment over a Black and White Planar Scene

**Figure 5:**
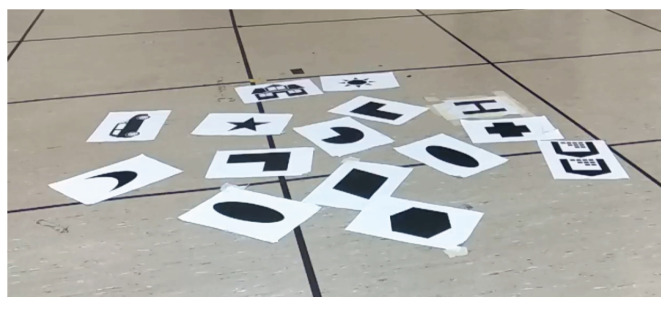
Scene used for the BW Planar experiments.

**Figure 6:**
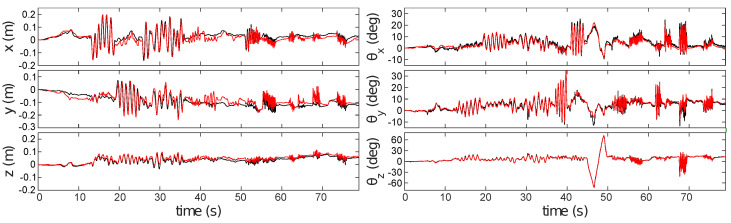
Comparison between pose estimates generated by ESPEE (red) and ground truth generated by the Vicon motion capture system (black). ESPEE is able to estimate the pose of the sensor.

The BW Planar test used a DAVIS240C with a 4.5 mm lens initialized at a height of 0.9 m. The scene is composed of printed shapes on white paper (Figure 5). The accurate planar assumption and strong black on white stimulus allow ESPEE to track the pose accurately (Figure 6) for fast movements (Appendix B). Still, some drifts can be observed in translation. This behavior can be explained by the thickness of some contrasts in the map, which does not allow an accurate pixel-matching and create errors in pose estimation. An ambiguity between rotation and translation is also observed and difficult to overcome in a planar scene.

This first test provides an assessment of the performance of the algorithm when the assumption of the planar scene is true. However, these conditions, flat scene and strong stimuli, are not an accurate representation of the real world; thus, the following test is more representative of what could be expected in a real scenario.

#### 3.1.2. Handheld Experiment over Various Objects

**Figure 7:**
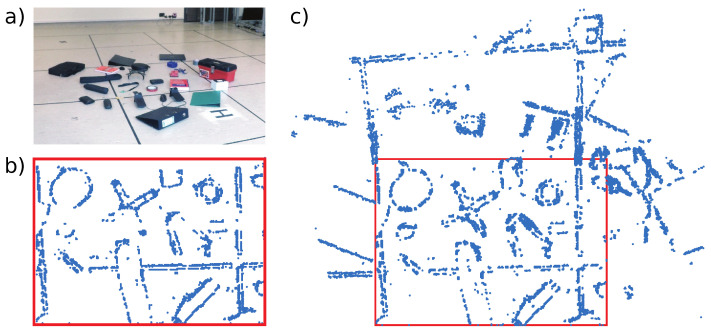
The scene used for the Objects test. (**a**) Frame capture of the scene consisting of various objects on the ground. (**b**) The feature map generated at initialization. Blue dots show features. The red box shows the sensor FoV. (**c**) The final map after the recording. New features have been added both inside and outside the initialization FoV.

Figure 7 shows the environment for the Objects test. A handheld DAVIS240C sensor was used with a 4.5 mm lens and initialized at a height of 1.2 m above the scene shown in Figure 7a. Figure 7b shows the feature map after initialization. New features are added to the map as ESPEE runs, and the final map is shown in Figure 7c. The final map extends the original map but also includes new features within the original Field of View (FoV) of the sensor (indicated by the red box).

**Figure 8:**
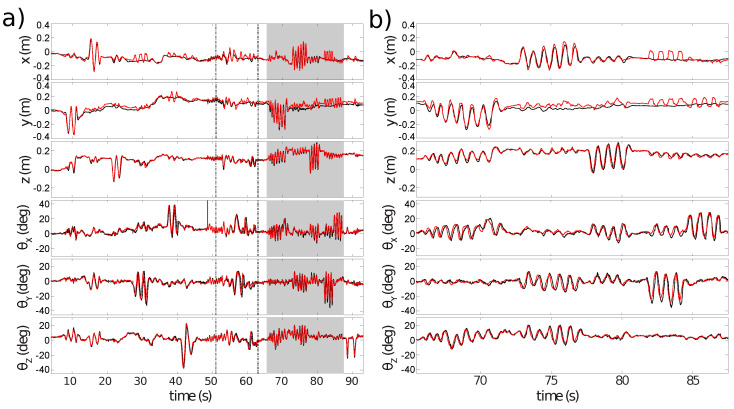
Comparison between pose estimates generated by ESPEE (red) and ground truth generated by the Vicon motion capture system (black). (**a**) The full 93 s sequence. Vertical lines indicate where room lighting was switched off (51 s) and on again (63 s). The shaded region shows a period of rapid motion which is magnified in (**b**). ESPEE performs well, but ambiguities between rotations and translations are visible between 82 and 87 s.

Figure 8 compares pose estimates generated by ESPEE (red) against pose measurements made with the Vicon motion capture system (black). Figure 8a shows the full sequence of 93 s. The two vertical lines indicate when lighting conditions changed by turning the room lights off and on again. ESPEE handled the lighting change without trouble. To be fair, it should be noticed that the action camera, a GoPro Hero 4, used to record the experiment was also able to adapt to the intensity change (see video as Supplementary Materials). Later testing in much darker conditions verified that ESPEE can still operate, although it could not handle as rapid motion under low lighting. It is most likely due to the increased jitter and reduced number of events in this condition, as it has been described in [35].

The shaded region of Figure 8a shows a period of rapid motion (magnified view in Figure 8b). During this period, the oscillation of each degree of freedom reaches the maximum speed, as indicated in Appendix B.

The pose continues to be estimated with good accuracy, but ambiguity between *y*-axis translation and *x*-axis rotation is visible (error in *y*-position from 82 s onwards). Similarly, there is ambiguity between *y*-axis rotation and *x*-axis translation (error in *x*-position between 82 and 85 s). These errors are proportional to the rotation magnitude and are exacerbated even further during large rotations by the fact that there will be reduced overlap between the FoV of the sensor and the mapped region of the scene; thus, increasing ambiguity between possible sensor poses.

#### 3.1.3. Handheld Experiment over Lines

**Figure 9:**
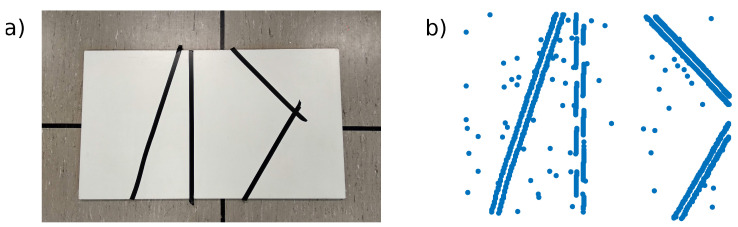
The scene used for the Lines test. (**a**) Frame capture of the scene consisting of various lines with few intersections. (**b**) The point cloud map generated at initialization with 1000 points in blue. The initial FoV was purposely did not contain any corners, and no new 3D points were added to the map during the test.

**Figure 10:**
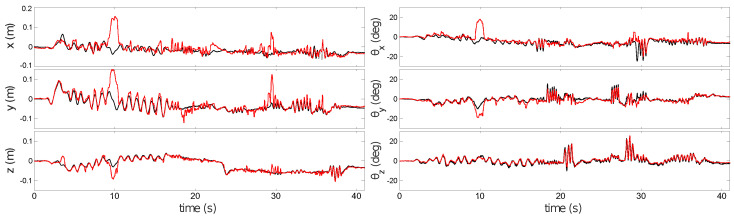
Comparison between pose estimates generated by ESPEE (red) and ground truth generated by the Vicon motion capture system (black). ESPEE still performs relatively well in a featureless scene consisting of only lines without crossings.

To demonstrate an advantage of the simplicity of the pixel to 3D point association algorithm. A test was performed with a cornerless environment consisting only of lines not crossing each other in the FoV. We used a white board with black tape to create the lines (see Figure 9a). The algorithm was initialized when no corners were visible (see Figure 9b), and was not updated later. As the scene was mostly uniform, the number of initialized points was reduced to 1000, which resulted in gaps in some edges, but limited the amount of 3D points unrelated to edges coming from noisy events perceived during initialization. Without any specific tuning, ESPEE was able to cope with this challenging environment, still providing accurate results (Figure 10). The estimated trajectory mostly follows the ground truth, except in the fast motion estimation. This behavior could be explained by the relative lack of features compared to the other examples. The lower event rate could also be a reason for these fast motion errors. One can argue that the lines are straight in this example. Indeed, the assumption of straight lines could make this problem easier. However, no such assumption has been made here; thus, curved lines could have also been used.

#### 3.1.4. Metrics

**Figure 11:**
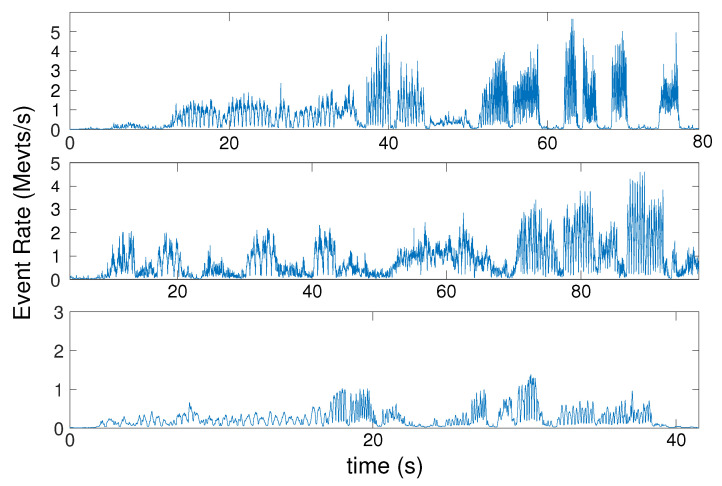
Event rates over time for each of the three tests. From top to bottom: BW planar, Objects, Lines.

**Figure 12:**
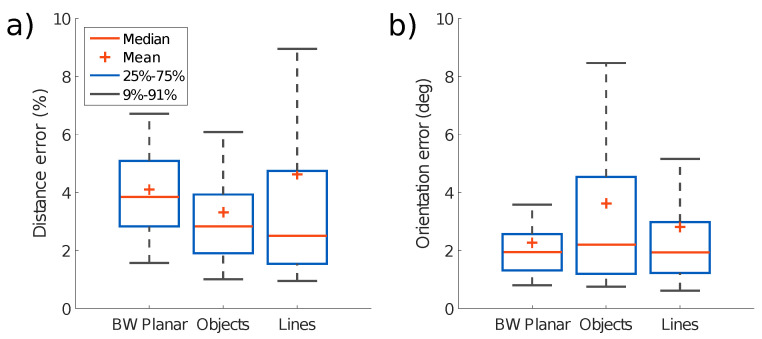
Absolute error statistics for the three test sequences presented. (**a**) Translational error as a percentage of scene depth. (**b**) Rotational error in terms of the geodesic distance between the estimated and the ground truth orientations.

In each test, the sensor is moved at different speeds. The speed of motion for each test is reported in Appendix B. Figure 11 shows the event rate over time for each scene, calculated as a moving average over 5k consecutive event intervals. For each test, we report the distribution of the errors in Figure 12. The statistical analysis is computed from the error between the estimation and the ground truth over time for each sample. Translation error is reported as a percentage of scene depth for each test. It can be seen that for all the tests, that the translation and orientation errors are on average below 5% of the mean depth and below 4${}^{\circ }$, respectively.

### 3.2. Event-Camera Dataset

**Figure 13:**
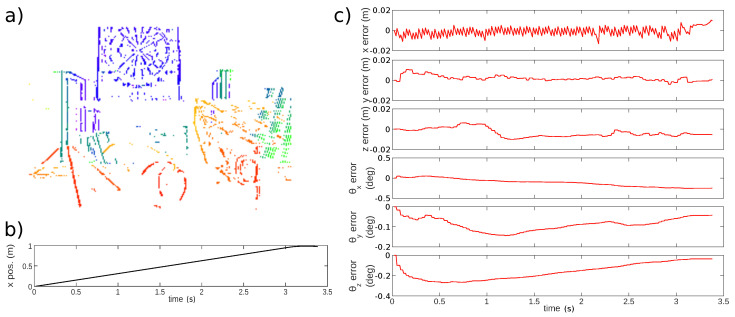
Localization performance on the *slider_depth* sequence. (**a**) Point cloud generated with EMVS code and example parameters. (**b**) Translation of the sensor along the *x*-axis while the other degrees of freedom are kept constant. (**c**) Errors of each degree of freedom (red).

To show the performance of the algorithm on more complex scenes, the algorithm was tested on some sequences of the event-camera dataset [36]. In order to make the algorithm work on this dataset, where the initialization is far from our initial pipeline, we used the point cloud generated by the EMVS provided thanks to the code available online (https://github.com/uzh-rpg/rpg_emvs, accessed on 25 November 2021). Therefore, in this configuration, it is only performing localization with a provided map, which is not a realistic scenario. However, we were able to display that the algorithm performs well on a 3D scene on the *slider_depth* example. Figure 13 reports the localization error for each degree of freedom.

### 3.3. Dual-Sensor Experiments

In the literature, multiple sensors usually have an overlapping field of view. Depth from stereo can then be used to provide scale to the environment [5]. However, exploiting multiple sensors can also bring more robustness and depth perception even without overlapping FoVs [37,38]. Here, we chose to place the two sensors orthogonally to easily discriminate rotation from translation, as it was a common confusion during the monocular experiments. This section displays the dual-sensor setup tested against larger depth differences and compared with monocular results.

**Figure 14:**
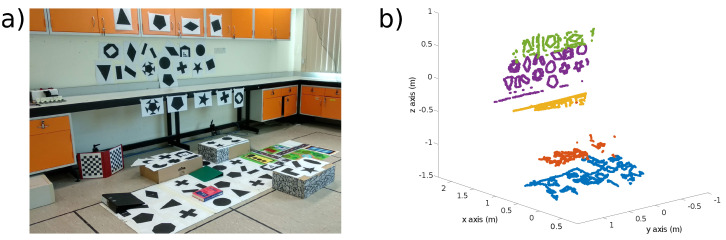
(**a**) Visual scene used for the dual-sensor experiment, with up to 60 cm difference in depth. (**b**) Point cloud obtained at time 13.5 s (i.e., after two keyframes have been triggered for each sensor) during the experiment with the dual sensor where the EMVS mapping is performed. The different colors represent the different planes contained in the scene.

Figure 14 shows the visual scene used for the experiment and the point cloud estimated from mapping. The boxes on the ground are between 17 and 20 cm high, where the lab bench edge and the closet are 60 and 30 cm from the wall, respectively.

**Figure 15:**
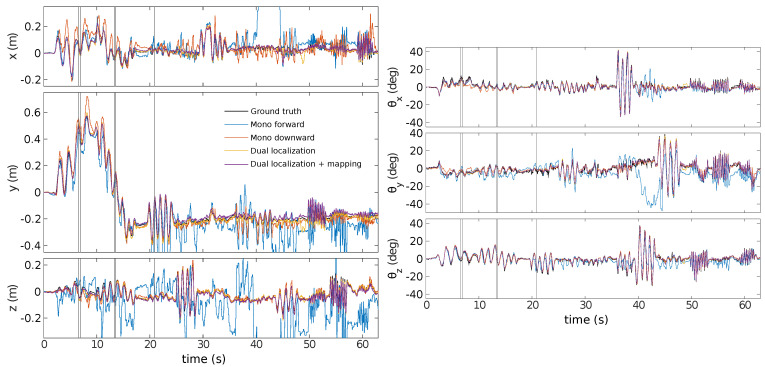
Pose estimates generated by ESPEE, the blue is the sensor in the forward direction, the red is the sensor in the downward direction, the yellow is both sensors with the planar scene assumption, the purple is with the EMVS mapping and in black ground truth generated by the Vicon motion capture system. Vertical lines in grey are the mapping updates during the Dual-sensor localization and mapping experiment.

The experiment uses handheld movements of the sensor along each degree of freedom individually before ending with rapid motion. Figure 15 displays the pose estimations for each sensor independently, for the dual estimation, all three using the planar scene assumption, and last, the dual sensor estimation with the mapping included. One can see that the estimate by the monocular sensor looking forward is far off at some point, as the rotation is mostly interpreted as translation. The FoV of the forward-facing sensor has the greatest variation in depth, leading to violation of the planar scene assumption, and thus, to errors in the state estimation.

**Figure 16:**
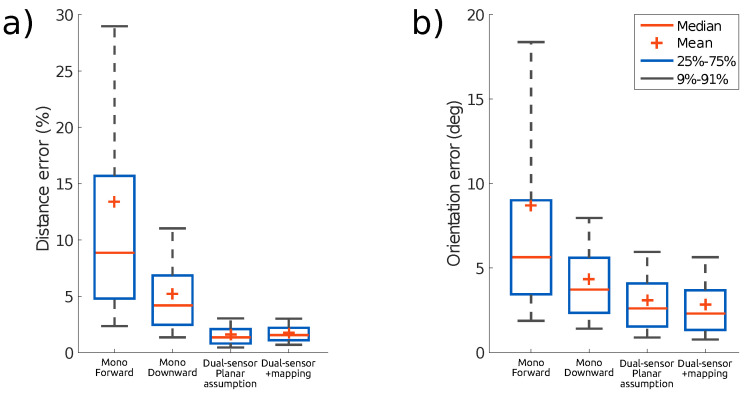
Statistical comparison of the results obtained with the dual-sensor experiment (Figure 15), with only one sensor pointing forward, only one sensor pointing downward, two sensors without any mapping updates, and two sensors with mapping updates, respectively. (**a**) Translation errors as a percentage of scene depth. (**b**) Rotational error in terms of the geodesic distance between the estimated and the ground truth orientations.

Figure 16 sums up the estimated pose error for each test and shows that the pose error is significantly reduced by using two sensors, with a greater improvement for the position estimation. This observation holds whether using the planar scene assumption or estimating depth from online mapping. The statistical results are computed similarly to Figure 12.

## 4. Discussion

The ESPEE algorithm presented in this paper displays an event-by-event 6-DoF pose estimation. The algorithm features a rather simple pixel to 3D point association step, by assuming all events are created by the closest point in the map, and by always keeping an up-to-date prediction of 3D point locations in a LUT. Such a LUT is easily updated at a rate of kHz on modern processors or on an FPGA.

ESPEE can provide pose estimates at rates over 1 MHz, even using an FPGA, but such high rates are not typically useful. In practice, multiple pose estimates could be averaged to provide fewer, less noisy estimates (smoothing is also used in [18]). Nevertheless, the high-speed, low-latency pose updates generated by ESPEE make it attractive for fast robotic applications.

Event rates for the high-speed BW Planar and Objects tests peak around 5 Mevts/s (Figure 11), exceeding the maximum sustained event rate for any of the processors tested (Table 1). Therefore, a temporary lag in generating the pose estimates during these peaks is expected, but the lag will disappear once the event rate drops again (if the buffer is not saturated, otherwise some events would be skipped).

The LUT is computed at fixed timesteps rather than at a fixed number of events. This choice was made with the FPGA implementation in mind as this LUT is updated as fast as possible in parallel with the pose estimation. Therefore, the latency in the LUT update is dependent on the number of 3D points, the FPGA clock speed, and the degree of parallelism used by the FPGA for projecting points onto the simulated image plane. Thus, when fast motion occurs, the LUT could be further away from the currently estimated pose (depending on the event rate), which would lead to an overestimated pose error and some small oscillations in the pose estimation once the new LUT is recomputed. Ideally, the LUT would be updated at each event, but it is not reasonable from an implementation perspective. Another approach could be to update the LUT at a fixed number of events, but it would lead to computation overhead during fast motion. Some approaches [18,39] apply motion compensation to incoming events to align them with a keyframe. A similar approach could be used to align events with the time of the last LUT update and relax the LUT update rate requirement. Another potential solution would be to gather the events between two LUT updates and generate a unique update of the EKF. Even if it would not be event-by-event anymore, it would still be difficult to parametrize the EKF as the prediction step should allow for a given displacement during a fixed period of time, which might not be consistent for all speeds. The proposed approach is a compromise between accuracy and latency.

Having a constant covariance matrix Q is a limitation regarding the robustness toward scene complexity. Indeed, given a constant event rate, if the scene contains a lower amount of contrasts, the average motion would be larger between each event compared to a richly textured scene. Thus, Q should be made larger to take into account the worst-case scenario to avoid being lost. However, it would result in a lower maximum speed in the richer environment. Having an inertial measurement could help to adapt Q, but special care should be taken to combine the rate-based IMU measurements to the event-based visual inputs (see [40,41] for examples).

The IMU could also help to provide the missing scaling factor. The initial depth allows the estimation of the pose up to scale, but constrains the initialization conditions. In the case of the dual-sensor, a wider baseline and a proper calibration could also be used, even if no FoV is shared, as shown in [38].

ESPEE works best on high contrast scenes with sharp edges, such as the BW Planar scene. Strong black on white contrasts provides strong signals, while sharp edges provide precise information for localization. Most natural scenes contain edges and thus are suitable for ESPEE. However, highly cluttered scenes will not work, as multiple candidates would be available in the event neighborhood for the pixel association. The randomized association would, therefore, be used most of the time, resulting in inaccurate pose estimation. Another challenging case would be a scene with intensity gradients and no clear edge. In this situation, the event would be triggered at inconsistent locations depending on the pixel adaptation level. Other algorithms, such as [27,29], attempt to track such intensity graduations at the expense of additional complexity.

In this paper, we show that ESPEE could be associated with the EMVS algorithm [22] as a means of acquiring the point cloud. However, the code published on Github has been modified to provide a fully event-by-event algorithm, which results in each event being associated with a unique pose. This approach was chosen to avoid buffering previous events or poses. We only show mapping with the dual-sensor setup because the robustness of the monocular setup was not sufficient to provide an accurate map for the full duration of a test without post-fitting and tuning the parameters.

The feature list used by ESPEE consists only of 3D points that generate events (typically points lying on edges). Occlusion is not modeled when these points are projected into the LUT used to match features, and therefore, events may be matched with occluded features that are not even visible from the current pose. The research in [18] is likely to suffer from a similar problem, but the world model used in [29] would account for occlusion. Discarding the old point cloud at each update should help with this problem, as well as prevent generating multiple 3D points in the map from the same point in the world.

## 5. Conclusions

We have presented ESPEE, an event-based algorithm for 6-DoF sensor pose estimation. The algorithm can handle fast motion and runs in real-time for the monocular setup, even on embedded hardware, such as FPGA. An extension to multiple sensors with non-overlapping FoVs improves robustness. This approach could benefit from an improved prediction process to allow better accuracy. Finding a way to compute local LUT efficiently could also be considered to achieve faster motion. This paper adds to a growing body of work showing the promise of event-based sensors for applications requiring low-latency sensing with limited computing resources, as is the case with aerial vehicles.

## Figures and Tables

**Table 1:** Average events throughput and processing time for the different functions.

Computing Device	Event Rate	Pixel-3D Point	EKF Processing	LUT Update
	(Mevts/s)	Assoc. (μs/evt)	(μs/evt)	(μs/3500 pts)
Intel Core i7 (4th gen.)	2.3	0.238	0.179	233
Odroid XU4	0.6	1.324	0.103	318
Spartan 6 l × 150	1.5	0.145	0.510	310

## Data Availability

The C++ code is available at https://github.com/fabien-colonnier/espee_ros, accessed on 25 November 2021.

## Supplementary Materials

The videos of the experiments presented in the paper can be found at the following links https://www.mdpi.com/article/10.3390/s21237840/s1. The first video named mono_exp.mp4 displays the monocular experiments “BW Planar”, “Objects”, and “Lines”, as well as the *slider_depth* sequences of the event-camera dataset [36]. The second video named dual_sensor_ compressed.mp4 shows the comparison between monocular and dual-sensor localization.

## Appendix A. EKF Parameters

The EKF parameters used for the experiments were determined empirically and are summed up in Table A1.

**Table A1:** EKF parameters used in the different experiments. diag means a square matrix where all non-zero-values are on the diagonal and presented in a vector form.

Sensor Setup	EKF Parameters
Monocular sensor exp.	$P=10^{-6}\times diag\left(1,1,1,0.03,0.03,0.03\right)$
	$Q=10^{-9}\times diag\left(5,5,5,30,30,30\right)$
	$R=diag\left(25*pixelsize/f,25*pixelsize/f\right)$
Dual sensor exp.	$P=10^{-6}\times diag\left(1,1,1,0.03,0.03,0.03\right)$
	$Q=10^{-9}\times diag\left(20,20,20,150,150,150\right)$
	$R=diag\left(200*pixelsize/f,200*pixelsize/f\right)$

## Appendix B. Motion Statistics

Table A2 displays the maximum speed registered for each degree of freedom in each test recording. As a reminder, the mean depths were 0.9, 1.2, 0.5, and 1.6 m for “BW Planar”, “Objects", “Lines”, and “Dual-Sensor”, respectively. This information is important to calculate the optic flow experienced.

**Table A2:** The maximum speed registered from the largest oscillations present in each of the test recordings.

	Translation (m/s)			Rotation (${}^{\circ }$/s)		
	$\dot{x}$	$\dot{y}$	$\dot{z}$	${\dot{\theta }}_{x}$	${\dot{\theta }}_{y}$	${\dot{\theta }}_{z}$
BW Planar	1.9	2.1	-	518	471	1016
Objects	2.3	2.4	2.7	349	283	212
Lines	1.6	1.3	1.9	647	764	677
Dual sensor	7.4	6.9	8.6	965	1022	879
